# Effect of interval exercise versus continuous exercise on excess post-exercise oxygen consumption during energy-homogenized exercise on a cycle ergometer

**DOI:** 10.20463/jenb.2019.0016

**Published:** 2019-06-30

**Authors:** Won-Sang Jung, Hyejung Hwang, Jisu Kim, Hun-Young Park, Kiwon Lim

**Affiliations:** 1 Physical Activity and Performance Institute (PAPI), Konkuk University, Seoul Republic of Korea; 2 Department of Physical Education, Konkuk University, Seoul Republic of Korea

**Keywords:** continuous exercise, interval exercise, excess post-exercise oxygen consumption (EPOC), energy expenditure

## Abstract

**[Purpose]:**

The purpose of this study was to confirm that the difference in excess post-exercise oxygen consumption (EPOC) during exercise of the spending the same calories between the continuous and interval exercise.

**[Methods]:**

Thirty-four healthy college students who did not regularly exercise volunteered to participate in our study. Continuous exercise was performed on an ergometer for 30 min at 60% of maximal oxygen consumption (VO_2 max_). Interval exercise was performed on a cycle ergometer at 80% VO_2 max_ for 2 min initially, followed by 40% VO_2 max_ for 1 min, and 80% VO_2 max_ for 3 min. This was repeated six times for a total of 26 min.

**[Results]:**

The major findings were as follows: (1) energy consumption during exercise was not significantly different between continuous exercise and interval exercise groups; (2) EPOC was higher in interval exercise than in continuous exercise for all dependent variables (i.e., total oxygen consumption, total calories, summation of heart rate); and (3) there were no significant differences in the lipid profile between continuous and interval groups.

**[Conclusion]:**

Our study confirmed that after equalizing energy expenditure for continuous and interval exercise on a cycle ergometer in subjects in their twenties, interval exercise results in higher EPOC than continuous exercise. These data suggest that interval exercise may be more effective than continuous exercise in reducing body fat, for a given amount of energy expenditure.

## INTRODUCTION

Many studies have demonstrated the importance and effectiveness of exercise in managing health and losing weight. The American College of Sports Medicine (ACSM) recommends an exercise intensity of 40–85% heart rate reserve (HRR) or oxygen uptake reserve (VO_2_R), a target energy expenditure of 150–400 kcal (or 20–60 min), and over three per weeks, 30 min of continuous exercise^1^.

The reported positive effects of continuous exercise include relatively high increases in blood levels of epinephrine, norepinephrine, and growth hormone, the increased use of fat as an energy source, and the secretion of insulin and cortisol^2,3,4^. Continuous exercise has also been reported to be effective for weight loss by increasing daily energy consumption^5,6^. However, despite these positive effects, not many people are able to maintain these habits due to time constraints, exercise intolerance, and monotony^7^. Interval training has been recommended as a new exercise method that can eliminate these shortcomings^8,9^. Interval training is a form of exercise in which short periods of intense exercise are alternated with less-intense recovery periods. It is good for improving both aerobic and anaerobic energy systems, and is very effective at increasing an individual’s VO_2 max_ and anaerobic threshold^10^. Thus, it is one of the most effective ways to improve cardiopulmonary functions, metabolic functions, health, and weight loss in the general population and in athletes^8^.

During vigorous and high intensity interval exercise, metabolic rates can increase exponentially, and the intensity and duration of exercise can greatly affect metabolic reactions, both during and after exercise^6^. In particular, during recovery, excess post-exercise oxygen consumption (EPOC) is used to restore the body to a resting state, and to adapt it to the exercise just performed. Several mechanisms are attributed to EPOC, such as replenishment of oxygen stores in muscle and blood, increased circulation and lactate removal, resynthesis of adenosine triphosphate (ATP) and creatine phosphate (CrP), increased triglyceride/fatty acid cycling, and increased heart rate (HR), ventilation, and body temperature^11,12,13^. 

Several studies have stated the importance of EPOC to continuous exercise and interval exercise, and EPOC size have suggested more continual of exercise at higher intensity^6,8,14^. However, in previous studies, most of the calories homogenized between continuous exercise and interval exercise were mainly compared to the same exercise time and different exercise intensity, or the amount of exercise estimated using a calculation formula. The was difference in actual calorie when the calories were homogenized with absolute intensity and time during exercise and there was no difference in EPOC^16^. Therefore, identifying the effect of EPOC on the homogenization of energy consumption between continuous exercise and interval exercise will be important in determining the optimal exercise routine to promote health and weight loss in the future. For accurate homogenization of kinetic energy consumption, it is important to eliminate extrinsic variables that affect the accuracy of VO_2 max_ measurements and EPOC measruements^14^. EPOC has been shown to be influenced by training status, exercise intensity and duration, and the thermic effect of food^6,15^. Thus, to make a direct comparison between recovery oxygen consumption after continuous and interval exercise, it is important to ensure that exercise variables such as total work and duration are as similar as possible and to avoid confounding factors such as food intake before and after exercise^14,16^.

Therefore, in this study we measured EPOC in continuous and interval exercise during or after exercise. Participants were provided with the same pre-food intake, and we homogenized the energy expenditure of during exercise between exercise types, in order to minimize extra variables, and accurately measure EPOC. In addition, the purpose of to provide was prescription of exercise by verifying the effect of EPOC of the continuous and interval exercise in subjects with twenties without exercise experience. The purpose of the present study was to confirm that the difference of EPOC during exercise of the spending the same amount of calories between the continuous and interval exercise.

## METHODS

### Participants

Thirty-four healthy college students in their twenties (mean age = 23.65 ± 2.17 years; n =18 men 16 women)’ who did not exercise regularly volunteered to participate in the study. Subjects who met one or more of the following exclusion criteria were deemed not eligible and were excluded from the study: unstable angina, having had a cardiac infarction within the previous four weeks, uncompensated heart failure, severe valvular illness, pulmonary disease, uncontrolled hypertension, kidney failure, orthopedic/neurological limitations, cardiomyopathy, planned surgery during the research period, reluctance to sign the consent form, drug or alcohol abuse, or involvement in another study. All subjects were fully acquainted with the nature of the study and were informed of the experimental risks before signing a written consent form. It was explicitly stated to the subjects that they could withdraw from the study at any point. All subjects had their pre-test research fully explained to them and provided voluntary consent. All procedures of the study was approved by the Institutional Review Board of Konkuk University (7001355-201903-HR-305) in Korea and was conducted according to the Declaration of Helsinki. 

### Experimental design

To test EPOC and energy expenditure during and after continuous and interval exercise, we used a balanced repeated measures crossover design. This approach required gathering data on the subjects’ completion of two training sessions on separate test days, in a randomized order.

Each participant visited the laboratory three times. On the first visit we performed body composition tests (InBody 770, Biospace Ltd, Seoul, Korea), and a maximal cardiopulmonary exercise test (Quark CPET, Cosmed, Italy) to determine the maximal values of VO_2_ (VO_2 max_). On the second and third visits, at 72 h after performing the maximal CPETs, respectively, individuals performed continuous cycle ergometer exercise at 60% of VO_2 max_, and interval cycle ergometer exercise at 40% or 80% of VO_2 max_. As soon as the exercise ended, subjects came down from the cycle ergometer, sat on a chair, and measured EPOC for 60 min.

### Pre-testing measurements

All subjects performed a maximal aerobic exercise test using a cycle ergometer (Aerobike, Combi 75 XL, Tokyo, Japan) in order to determine their VO_2 max_. The work rate at 50 rpm was 50 W for men and 25 W for women for the first 2 min, and was increased by 25 W for men and 12.5 W for women every 2 min. This continued either until exhaustion or until subjects were unable to maintain 50 rpm. The criteria for having reached the true VO_2 max_ was showing a plateau in VO_2_ uptake, despite increased intensity of exercise and a respiratory exchange ratio (RER) above 1.15. HR was monitored using a Polar 800 device (Polar Electro, Kempele, Finland).

### Exercise training protocol

Participants were transported to the laboratory at 8 am after a 12-h fast and 48-h abstention from vigorous physical activity. They were given a standardized breakfast of 2 pieces of bread (200 kcal), 1 boiled egg (80 kcal), 1 cup of orange juice (120 kcal), and 1 cup of water. Subjects rested in a comfortable posture after breakfast and participated in the experiment 2 h later. Ambient room temperature was maintained at 23 ± 1 °C. After 10 min of quiet sitting as a habituation period, we measured VO_2_, ventilation, and RER for 5 min. The average was used as the baseline (BASE). The subjects then performed continuous or interval exercise on a cycle ergometer (Aerobike, Combi 75 XL, Tokyo, Japan). Speed was adjusted on an individual basis, according to each subject’s fitness level. The continuous exercise training was performed for 30 min at 60% of VO_2 max_, and the interval exercise training was performed first for 2 min at 80% of VO_2 max_, followed by 1 min at 40% of VO_2 max_, and finally for 3 min at 80% of VO_2 max_. This was repeated six times for a total of 26 min. The calories expended between the continuous exercise (Con Ex) and interval exercise (Inter Ex) groups were not statistically different (212.24 ± 68.47 vs 214.85 ± 66.32, p=0.503).

### EPOC measurement

Immediately after exercise, participants were seated in a chair and relative VO_2_, absolute VO_2_, Kcal, HR, and duration were monitored for 60 min. EPOC values were determined at the time when VO_2_, HR, and RER values returned to the resting baseline. Collection and analysis of lipid samples was done before exercise, immediately after exercise, after 30 min and after 60 min. Total cholesterol (TC), triglyceride (TG), high-density lipoprotein (HDL) cholesterol, and low-density lipoprotein (LDL) cholesterol were measured using a portable digital lipid analyzer (SD LipidoCare, SD Biosensor, Inc., Seoul, Korea).

### Statistics

All statistical analyses were completed using IBM SPSS Statistics 23 (SPSS Inc., Chicago, IL, USA). Data normality was verified using the Shapiro-Wilk test, and descriptive data are presented as mean ± standard deviation. A paired t-test was used to compare the differences between the two protocols. The effects of condition on EPOC were analyzed using a mixed procedure. Where main effects were statistically significant, post-hoc pairwise comparisons with Sidak-adjusted p-values were performed. Model-fitting was evaluated using Hurvich and Tsai’s criteria. All statistical assumptions were checked using standard graphical procedures. Statistical significance was accepted for p<0.05.

## RESULTS

**Figure 1.**
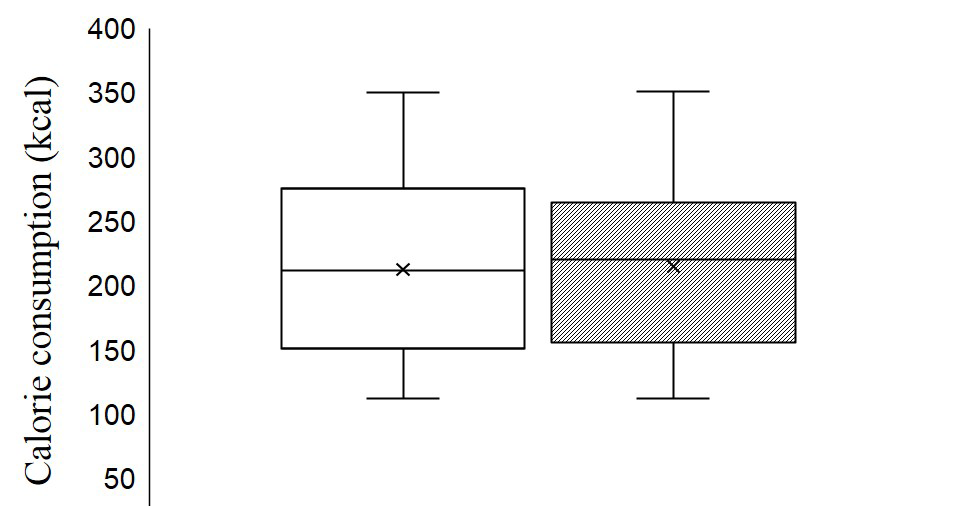
Comparison of oxygen consumption during exercise

Figure 1 shows that the amount of calories expended during exercise was not significantly different between continuous and interval exercise(p=0.503).

Based on the EPOC results shown in Table 2, the EPOC duration was longer for interval exercise than in continuous exercise (31.24 ± 15.09 vs 45.90 ± 12.37, p < .001), which showed higher Inter Ex than in the Con Ex in all variables including VO_2_ total (11992.40 ± 6481.05 vs 17425.24 ± 6329.98, p<0.001; men: 14980.78 ± 6529.74 vs 21410.32 ± 5411.21, p=0.001; women: 8630.48 ± 4616.70 vs 12942.03 ± 3803.88, p<0.001), VO_2_/kg total (185.42 ± 98.94 vs 266.81 ± 79.62, p<0.001; men: 204.83 ± 103.40 vs 289.68 ± 84.35, p=0.001; women: 163.59 ± 91.98 vs 241.08 ± 67.46, p=0.001), Kcal total (58.14 ± 31.42 vs 82.72±28.69, p<0.001; men: 72.80 ± 31.82 vs 100.96 ± 23.79, p=0.004; women: 41.65 ± 21.82 vs 62.21 ± 17.92, p = 0.001) and HR sum (2931.64 ± 1560.92 vs 4557.10 ± 1419.05, p<0.001; men: 3026.21 ± 1346.65 vs 4630.17 ± 1330.58, p<0.001; women: 2825.24 ± 1811.68 vs 4474.89 ± 1552.43, p=0.001). When the results of oxygen-deficient were examined, VO_2_ total (594.11 ± 242.10 vs 721.90 ± 347.90 p=0.009; men: 729.80 ± 194.71 vs 916.99 ± 347.18, p=0.006; women: 441.71 ± 197.76 vs 502.42 ± 180.62, p=0.394) showed a greater value than con Ex in inter Ex, and after separating the results for men and women, significant differences were only found in men. There was no significant difference in HR sum levels.

**Table 1.** Participant characteristics. Data represent the mean ± SD

Variable	Men (n=18)	Women (n=16)	Total (n=34)
Age (years)	24.28±2.49	22.94±1.53	23.65±2.17
Height (cm)	177.43±7.78	159.48±4.30	168.98±11.06
Weight (kg)	75.38±9.98	53.88±6.10	65.26±13.67
BMI (kg/m2)	23.86±2.04	21.19±2.28	22.61±2.52
Lean body mass (kg)	61.11±8.14	37.17±2.84	49.84±13.60
Fat mass (kg)	14.27±5.30	16.71±4.42	15.41±4.99
% fat mss (%)	18.74±5.71	30.01±6.06	24.05±8.13
VO_2max_ (mL/min/kg)	36.84±6.16	41.08±4.49	32.08±3.86

*Note*: SD = standard deviation, BMI = body mass index.

**Table 2.** Comparison of EPOC in Con EX vs Inter EX, ± SD

Variables		EPOC				O_2_ Deficit		
		VO_2__total (mL/min)	VO_2_/kg_total (mL/min/kg)	Kcal_total (kcal/min)	HR_sum	VO_2__total (mL/min)	Kcal_total (kcal/min)	HR_sum
Con Ex		11992.4±6481.05	185.42±98.94	58.14±31.42	2931.64±1560.92	594.11±242.10	3.39±1.35	28.98±8.17
Inter Ex		17425.24±6329.98	266.81±79.62	82.72±28.69	4557.1±1419.05	721.9±347.90	3.88±1.9	29.63±10.81
Δ%		45.3	43.89	42.28	55.45	21.51	14.45	2.24
Sig (p)		.000***	.000***	.000***	.000***	.009**	0.066	0.747
Men	Con Ex	14980.78±6529.74	204.83±103.40	72.8±31.82	3026.21±1346.65	729.8±194.71	4.19±1.06	29.00±8.74
	Inter Ex	21410.32±5411.21	289.68±84.35	100.96±23.79	4630.17±1330.58	916.99±347.18	4.94±1.96	30.73±13.36
Δ%		42.92	41.42	38.68	53	25.65	17.9	3.82
Sig (p)		.001**	.001**	.004**	.000***	.006**	0.066	0.758
Women	Con Ex	8630.48±4616.70	163.59±91.98	41.65±21.82	2825.24±1811.68	441.71±197.76	2.48±1.04	28.28±7.74
	Inter Ex	12942.03±3803.88	241.08±67.46	62.21±17.92	4474.89±1552.43	502.42±180.62	2.69±0.89	28.4±7.19
Δ%		49.96	47.37	49.36	58.39	13.74	8.47	0.42
Sig (p)		.001**	. 001**	.001**	.001**	0.394	0.557	0.938

*Note*: SD = standard deviation, Con Ex = continuous exercises, Inter Ex = Interval exercise, EPOC = excess post-exercise oxygen consumption, O_2_ = Oxygen , VO_2_ = oxygen consumption, HR = heart rate , Sum = summation, * p<.05, ** p<.01, *** p<.001.

**Figure 2.**
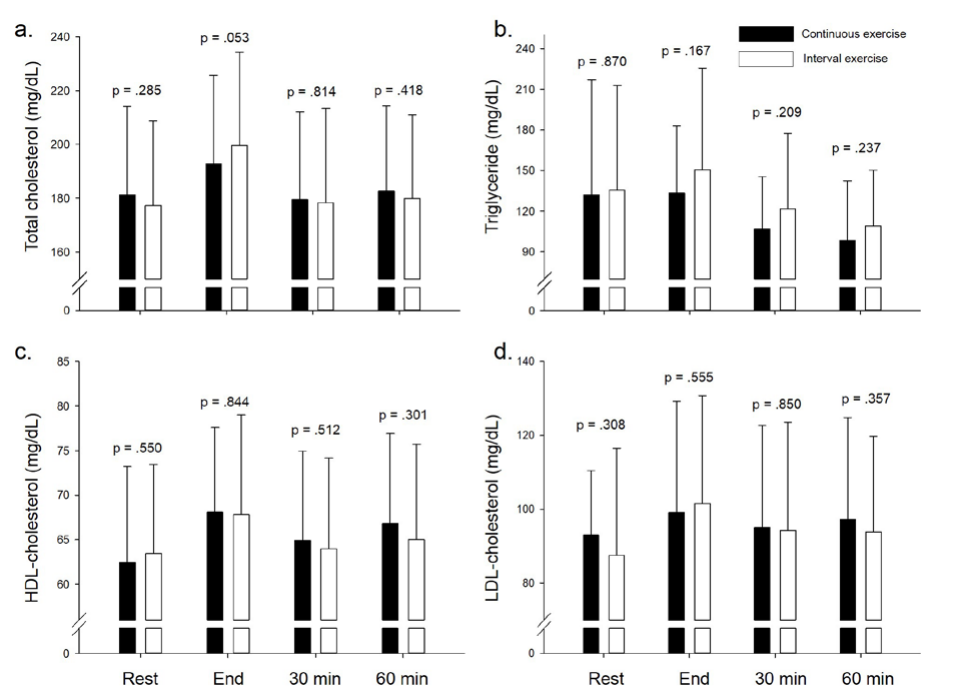
Comparison of lipid profile on EPOC in Con EX vs Inter EX

Figure 2 is a comparison of lipid profiles on EPOC in continuous and interval exercise. There were no significant differences in total cholesterol, triglyceride, HDL- cholesterol, or LDL- cholesterol in all variables (p>0.05).

## DISCUSSION

The purpose of this study was to confirm that there is a difference in excess post-exercise oxygen consumption (EPOC) between continuous and interval exercise, when expending the same number of calories. The major findings were: (1) energy consumption during the exercise was not significantly different between continuous exercise and interval exercise, (2) EPOC was higher in interval exercise than in continuous exercise for all dependent variables (e.g. total oxygen consumption, total calorie, and summation of heart rate), and (3) there was no significant differences in lipid profiles.

In previous studies that did not homogenize energy consumption during exercise, EPOC was higher in the interval exercise compared to continuous exercise and interval exercise^17-20^. In addition, Williams et al.^21^ compared the EPOC of 20 min of high intensity interval exercise and 60 min of continuous exercise, and found that the EPOC 30 min after exercise was higher in interval exercise, but the total EPOC after exercise was higher in continuous exercise. Larsen et al.^11^ reported that with increasing intensity, EPOC and EPOC duration increase, but if interval times are shorter, EPOC is reduced to similar levels as seen in continuous exercise. Tucker et al.^18^ showed that in high-intensity interval exercise oxygen consumption was low, but EPOC was high. However, summation of oxygen consumption during exercise and EPOC was higher in continuous exercise. As such, when did not homogenize energy consumption of exercise in EPOC results show that the interval exercise is more effective, but it is difficult to suggest that the effect of the interval exercise is effective when the total exercise energy consumption is not significantly different. By difference energy consumption of exercise in the continuous and interval exercise resulted in higher initial EPOC in the interval exercise but higher total energy consumption in the continuous exercise, so ensuring equivalence between the exercise is considered important. Thus, it our data suggest that the equalization of calories during exercise is an important factor in determining EPOC and is an important factor to consider in presenting the effects of exercise. In this study, we consider EPOC to have been significantly increased, because caloric expenditure was well-controlled during food-intake and exercise.

In a study that homogenized energy consumption between continuous and spaced movements, McGarvey et al.^16^ reported no significant differences in EPOC between 31 min of continuous exercise at 65% of VO_2 max_, and an interval exercise pattern of 90% VO_2 max_ for 2 min followed by 30% VO_2 max_ for 3 min, repeated 7 times for a total of 35 min. This may reflect differences in the EPOC measurement method. Most of the increase in oxygen consumption after exercise occurs in the early stages of recovery. As recovery continues, oxygen consumption decreases drastically, and the size increase with increasing standardized-duration decreases. Therefore, it is necessary to end when VO_2_, HR, and RER return to the baseline. In addition, the method to homogenize energy consumption during exercise and EPOC was performed well.

In our study, interval exercise resulted in post-exercise VO_2_, kcal, HR and EPOC measures 40% higher than for continuous cycle ergometer exercise. The results of this study support the hypothesis that the magnitude of EPOC and its duration is primarily dependent on exercise intensity^6,14^. In relation to the increase in EPOC, the ‘Oxygen Debt’ theory may explain this finding. For example, it could be explained by the energy cost to resynthesize glycogen from lactate, the exercise-induced increase in core temperature, the resynthesis of ATP/CP stores, and changes in cytokine release^20,22^. Consequently, greater exercise intensity may further increase the oxygen deficit at the onset of exercise, thereby affecting the body's homeostatic nature and resulting in a larger post-exercise O_2_ intake. Mechanisms responsible for this could extend to increases in VO_2_^6,23^. As shown in Table 2 of our study, the increase in oxygen deficit increased by more than 20% for interval exercise, as compared with continuous exercise. These results are therefore consistent with previous studies that show increased oxygen consumption during recovery after high intensity interval exercise, because of increased oxidative metabolism that supplements energy expenditure after exercise^24-26^.

In conclusion, our study confirmed that after homogenizing the energy expenditure of continuous and interval exercise on a cycle ergometer, EPOC is higher in interval exercise than continuous exercise in subjects who are in their twenties. This observation is important as it may help us understand why interval exercise has a greater propensity to induce weight loss than continuous exercise. Furthermore, these data provides a metabolic basis for enhanced fat loss during interval training that will be useful in establishing public health guidelines on exercise recommendations and weight management practices to reduce body fat. This should be qualified as only appropriate for young and healthy older populations who can perform such exercises. These exercise recommendations may promote weight loss and health, and result in better health outcomes in "time poor" modern lifestyles. Consequently, we suggest that interval exercise may be a more effective strategy in reducing body fat for energy expenditure increase than continuous exercise.

## References

[1] American College of Sports Medicine (2018). ACSM's guidelines for exercise testing and prescription.

[2] Ramos JS, Dalleck LC, Tjonna AE, Beetham KS, Coombes JS. (2015). The impact of high-intensity interval training versus moderate-intensity continuous training on vascular function: a systematic review and meta-analysis. *Sports Med.*.

[3] Peake JM, Tan SJ, Markworth JF, Broadbent JA, Skinner TL, Cameron-Smith D. (2014). Metabolic and hormonal responses to isoenergetic high-intensity interval exercise and continuous moderate-intensity exercise. *Am J Physiol Endocrinol Metab.*.

[4] Daly W, Seegers CA, Rubin DA, Dobridge JD, Hackney AC. (2005). Relationship between stress hormones and testosterone with prolonged endurance exercise. *Eur J Appl Physiol.*.

[5] Martins C, Stensvold D, Finlayson G, Holst J, Wisloff U, Kulseng B, Morgan L, King NA. (2015). Effect of moderate-and high-intensity acute exercise on appetite in obese individuals. *Med Sci Sports Exerc.*.

[6] Børsheim E, Bahr R. (2003). Effect of exercise intensity, duration and mode on post-exercise oxygen consumption. *Sports Med.*.

[7] Cunha FA, Midgley AW, McNaughton LR, Farinatti PT. (2016). Effect of continuous and intermittent bouts of isocaloric cycling and running exercise on excess postexercise oxygen consumption. *J Sci Med Sport.*.

[8] Weston KS, Wisløff U, Coombes JS. (2014). High-intensity interval training in patients with lifestyle-induced cardiometabolic disease: a systematic review and meta-analysis. *Br J Sports Med.*.

[9] Laursen PB, Jenkins DG. (2002). The scientific basis for high-intensity interval training: optimising training programmes and maximising performance in highly trained endurance athletes. *Sports med.*.

[10] Guiraud T, Nigam A, Gremeaux V, Meyer P, Juneau M, Bosquet L. (2012). High-intensity interval training in cardiac rehabilitation. *Sports med.*.

[11] Larsen I, Welde B, Martins C, Tjønna AE. (2014). High-and moderate-intensity aerobic exercise and excess post-exercise oxygen consumption in men with metabolic syndrome. *Scand J Med Sci Sports.*.

[12] Mooren FC (2012). Excess Postexercise Oxygen Consumption. Encyclopedia of Exercise Medicine in Health and Disease.

[13] Short KR, West JM, Sedlock DA. (1996). The effect of upper body exercise intensity and duration on post-exercise oxygen consumption. * Int J Sports Med.*.

[14] LaForgia J, Withers RT, Gore CJ. (2006). Effects of exercise intensity and duration on the excess post-exercise oxygen consumption. * J Sports Sci.*.

[15] Schaun GZ, Alberton CL, Ribeiro DO, Pinto SS. (2017). Acute effects of high-intensity interval training and moderate-intensity continuous training sessions on cardiorespiratory parameters in healthy young men. *Eur J Appl Physiol.*.

[16] McGarvey W, Jones R, Petersen S. (2005). Excess post-exercise oxygen consumption following continuous and interval cycling exercise. *Int J Sport Nutr Exerc Metab.*.

[17] Schaun GZ, Pinto SS, Praia ABC, Alberton CL. (2018). Energy expenditure and EPOC between water-based high-intensity interval training and moderate-intensity continuous training sessions in healthy women. *J Sports Sci.*.

[18] Tucker WJ, Angadi SS, Gaesser GA. (2016). Excess postexercise oxygen consumption after high-intensity and sprint interval exercise, and continuous steady-state exercise. *J Strength Cond Res.*.

[19] Gerber T, Borg ML, Hayes A, Stathis CG. (2014). High-intensity intermittent cycling increases purine loss compared with workload-matched continuous moderate intensity cycling. *Eur J Appl Physiol.*.

[20] Townsend JR, Stout JR, Morton AB, Jajtner AR, Gonzalez AM, Wells AJ, Mangine GT, McCormack WP, Emerson NS, Robinson EH, Hoffman JR, Fragala MS, Cosio-Lima L. (2013). Excess post exercise oxygen consumption (EPOC) following multiple effort sprint and moderate aerobic exercise. *Kinesiology*.

[21] Williams CB, Zelt JG, Castellani LN, Little JP, Jung ME, Wright DC, Tschakovsky ME, Gurd BJ. (2013). Changes in mechanisms proposed to mediate fat loss following an acute bout of high-intensity interval and endurance exercise. *Appl Physiol Nutr Metab.*.

[22] Sedlock DA. (1992). Post-exercise energy expenditure after cycle ergometer and treadmill exercise. *J Strength Cond Res.*.

[23] Noordhof DA, de Koning JJ, Foster C. (2010). The maximal accumulated oxygen deficit method: a valid and reliable measure of anaerobic capacity?. *Sports Med.*.

[24] Sedlock DA, Lee MG, Flynn MG, Park KS, Kamimori GH. (2010). Excess postexercise oxygen consumption after aerobic exercise training. *Int J Sport Nutr Exerc Metab.*.

[25] Paoli A, Moro T, Marcolin G, Neri M, Bianco A, Palma A, Grimaldi K. (2012). High-Intensity Interval Resistance Training (HIRT) influences resting energy expenditure and respiratory ratio in non-dieting individuals. *J Transl Med.*.

[26] Hagberg JM, Mullin JP, Nagle FJ. (1980). Effect of work intensity and duration on recovery O2. *J Appl Physiol Respir Environ Exerc Physiol.*.

